# The value of comorbidities and illness severity scores as prognostic tools for early outcome estimation in patients with aneurysmal subarachnoid hemorrhage

**DOI:** 10.1007/s10143-022-01890-5

**Published:** 2022-11-11

**Authors:** Vesna Malinova, Tuzi Sheri, Beate Kranawetter, Onnen Moerer, Veit Rohde, Dorothee Mielke

**Affiliations:** 1 Department of Neurosurgery, University Medical Center, 37075 Göttingen, Germany; 2 Department of Neurosurgery, Georg-August-University, Robert-Koch-Straße 40, 37075 Göttingen, Germany; 3 Department of Anesthesiology, University Medical Center, Göttingen, Germany

**Keywords:** CT perfusion, Subarachnoid hemorrhage, Functional outcome, Delayed infarction

## Abstract

Aneurysmal subarachnoid hemorrhage (aSAH) is a severe cerebrovascular disease not only causing brain injury but also frequently inducing a significant systemic reaction affecting multiple organ systems. In addition to hemorrhage severity, comorbidities and acute extracerebral organ dysfunction may impact the prognosis after aSAH as well. The study objective was to assess the value of illness severity scores for early outcome estimation after aSAH. A retrospective analysis of consecutive aSAH patients treated from 2012 to 2020 was performed. Comorbidities were evaluated applying the Charlson comorbidity index (CCI) and the American Society of Anesthesiologists (ASA) classification. Organ dysfunction was assessed by calculating the simplified acute physiology score (SAPS II) 24 h after admission. Modified Rankin scale (mRS) at 3 months was documented. The outcome discrimination power was evaluated. A total of 315 patients were analyzed. Significant comorbidities (CCI > 3) and physical performance impairment (ASA > 3) were found in 15% and 12% of all patients, respectively. The best outcome discrimination power showed SAPS II (AUC 0.76), whereas ASA (AUC 0.65) and CCI (AUC 0.64) exhibited lower discrimination power. A SAPS II cutoff of 40 could reliably discriminate patients with good (mRS ≤ 3) from those with poor outcome (*p* < 0.0001). Calculation of SAPS II allowed a comprehensive depiction of acute organ dysfunctions and facilitated a reliable early prognosis estimation in our study. In direct comparison to CCI and ASA, SAPS II demonstrated the highest discrimination power and deserves a consideration as a prognostic tool after aSAH.

## Introduction

Aneurysmal subarachnoid hemorrhage (aSAH) is a severe cerebrovascular disease regularly accompanied by a systemic reaction and subsequently compromising not only the brain function but also the function of multiple organ systems, which in turn may influence the outcome of aSAH patients as well [5, 11, 26]. Considering this, aSAH rather represents a multisystemic disease requiring a comprehensive multimodal management for preservation of organ functions than a disorder restricted to the brain. The widely used aSAH grading scales are based on the amount of extravasated blood on initial imaging and the neurological impairment at admission. These scales allow to some degree a risk stratification for cerebral and extracerebral complications and provide an early prognosis estimation. However, there are high-grade aSAH patients with good outcome as well as patients with initially lower aSAH grade, who experience a poor outcome, indicating a need for further individual risk stratification to facilitate a more concise early prognosis estimation [7, 34, 37]. Several tools are currently available for evaluation of comorbidities and illness severity harboring the same goal setting but pursuing a slightly different approach. The Charlson comorbidity index (CCI) is one of the most frequently used comorbidity measures for systematic assessment of comorbidities, which has been reliably applied in different patient populations [4]. The American Society of Anesthesiologists (ASA) classification not only accounts for the presence of comorbidities but additionally considers the physical performance impairment by the comorbidities and is usually calculated during the preoperative patient assessment for risk estimation of general anesthesia [23, 28]. Furthermore, the systemic reaction induced by the aSAH can be captured by illness severity scores such as the simplified acute physiology score II (SAPS II), which is frequently used for fatality prediction in intensive care patients [2, 14]. While these scores have been widely evaluated in different intensive care unit (ICU) patient populations, their prognostic role in aSAH patients is still largely unexplored and no studies have been published so far directly comparing the performance of these scores for early outcome estimation within 24 h after admission with aSAH. The aim of this study was to assess whether there is a correlation of comorbidities, physical performance impairment, and early organ dysfunctions with in-hospital mortality and functional outcome in aSAH patients by applying the above-mentioned scores within 24 h after admission in a large consecutive patient population with aSAH. Additionally, a direct comparison of their discrimination power concerning in-hospital mortality and functional outcome at 3-month follow-up was performed. Furthermore, the contribution of illness severity score to the prognostic value of established aSAH grading like the World Federation of Neurosurgical Societies (WFNS) scale was evaluated.

## Materials and methods

### Patient population

A retrospective analysis of a consecutive patient cohort diagnosed and treated with aSAH between January 2012 and December 2020 was conducted. Only adult patients with confirmed SAH by cranial computed tomography (CCT) and computed tomography angiography (CTA) or digital subtraction angiography (DSA) proving the presence of an aneurysm were enrolled. Patients with non-aneurysmal SAH were excluded. All patients were treated at the ICU for at least 14 days. For detection of delayed ischemic complications, a predefined interdisciplinary (neurosurgery, anesthesiology, neuroradiology) management protocol was applied [19]. This protocol included a neurological assessment at least twice a day and measurement of blood velocity of the middle cerebral artery by transcranial Doppler sonography (TCD) on a daily base. Blood flow velocity over 120 cm/s (mean) was considered a relevant increase, which indicated further diagnostics. Angiographic vasospasm was diagnosed by CTA or DSA and defined as arterial narrowing of at least 50% compared to the vessel diameter on admission, whereas a vessel narrowing of > 75% was considered severe angiographic vasospasm. Neurological deterioration in course of the disease with new-onset focal neurological deficits or decrease in consciousness was considered delayed ischemic neurological deficits (DINDs) after other possible causes such as hydrocephalus, epileptic seizures, or electrolyte disturbances have been excluded [30]. The aneurysm occlusion by microsurgical clipping or endovascular coiling was performed within 48 h after ictus. A CCT was conducted within 24 h after aneurysm occlusion to exclude treatment-associated cerebral infarction. Other new-onset cerebral infarctions that have been diagnosed on the CCT later were considered delayed cerebral infarctions in association with delayed cerebral ischemia (DCI). For the confirmation of DCI, computed tomography perfusion (CTP) was performed in case of DINDs and in case of TCD vasospasm to detect perfusion deficits and to identify tissue at risk for developing delayed infarctions. In sedated or comatose patients, in whom neurological assessment was not possible, CTP was additionally performed on day 3 and on day 7 after ictus for prediction and detection of DCI [20]. The occurrence of delayed infarction in association with DCI was documented.

### Assessment of comorbidities and organ functions

The presence of comorbidities was evaluated by calculating the CCI as defined by Charlson et al. [4]. Data on the following comorbidities were extracted from the medical records: arterial hypertension, coronary heart disease, arrhythmia, cardiac insufficiency, smoking, diabetes mellitus, hyperlipidemia, obesity, alcohol consumption, drug abuse, renal insufficiency, liver insufficiency, pulmonary disease (i.e., chronic obstructive lung disease, bronchial asthma), malignancy (solid tumors or leukemia), dysfunction of the thyroid gland, depression, stroke, neurological disease (i.e., multiple sclerosis, myasthenia, polyneuropathy, Parkinson’s disease, epilepsy), or other diseases (chronic pain syndrome, osteoporosis, dermatologic diseases, polyarthritis, gastrointestinal diseases). The ASA classification has been published in 1941 for preoperative stratification of the physical status of the patients based on comorbidities [21, 27]. Six ASA classes have been defined: I (healthy patient), II (patient with mild systemic disease without functional limitations), III (patient with severe systemic disease and substantial functional limitations), IV (patient with severe systemic disease that is a constant threat of life), V (moribund patient), VI (a declared brain-dead patient). A more comprehensive assessment of the patient’s physical status can be performed with the SAPSII including age, vital signs (heart rate, systolic blood pressure, body temperature), oxygenation, electrolytes, urine output, bilirubin and serum urea, leucocyte count, the presence of chronic disease (metastatic cancer, hematologic malignancy or AIDS), and Glasgow Coma Scale (GCS) score, as well as the type of admission (medical, scheduled surgical or unscheduled surgical), which was originally developed to estimate the probability of in-hospital mortality of ICU patients [14].

### Primary outcome parameter

The in-hospital mortality and the functional outcome were the primary outcome parameters in the study. The functional outcome was assessed as modified Rankin scale (mRS) at 3-month follow-up after ictus. The outcome was documented in the medical records by the neurosurgeon, who examined the patient in the outpatient unit during the follow-up examination.

### Statistical analysis

The statistical analyses were performed by means of the GraphPad Prism software (Version 9, GraphPad Software, San Diego, CA, USA). For the presentation of baseline data, descriptive statistics, and frequency distribution, analysis was done. Continuous variables are depicted as mean ± standard deviation (SD) and categorical variables as frequency or percentages. Receiver operating curve (ROC) analysis was performed and the area under the curve (AUC) was calculated for all three scores for direct comparison of their discrimination power of in-hospital mortality as well for discrimination of good (mRS ≤ 3) and poor functional outcome (mRS > 3). Youden index was calculated to determine the cutoff value with best discrimination of good and poor outcome. Multivariate logistic regression analysis was performed to identify independent predictors of in-hospital mortality and functional outcome. Since GCS has been considered for the calculation of SAPS II and of WFNS grading, the contribution of GCS to the SAPS II overall score calculation was additionally evaluated.

## Results

### Patients’ characteristics

**Fig. 1:**
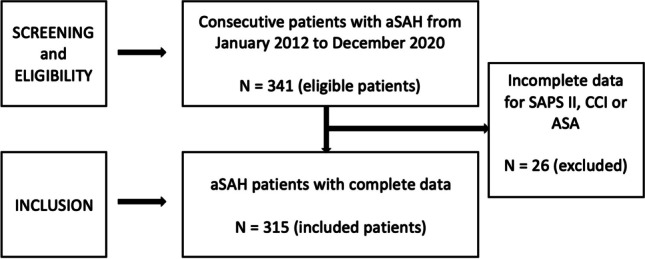
STROBE flow diagram of the eligible and included patients into the analysis of the study

A consecutive patient population consisting of 315 patients with aSAH was included into the analysis of this study. Due to incomplete data concerning SAPS II, CCI, and ASA, 26 patients were excluded (Fig. 1). Mean age of the patients was 55.6 years (range 23–90), a good WFNS grade (I–III) was found in 58.7% (185/315), and a high Fisher grade (3–4) was detected in 88.9% (280/315) of all patients. In 54% (170/315), the aneurysm was secured by microsurgical clipping and in 46% (145/315) by endovascular coiling. An overview of the patients’ characteristics is given in Table 1.

**Table 1:** Patients’ characteristics

Number of patients	315
Age in years	
- Mean	55.6 ± 13.9
- Median	55
- Range	23 - 90
Sex	
- Male	115 (63.5%)
- Female	200 (36.5%)
Aneurysm localization	
- Anterior circulation	268 (85.1%)
- Posterior circulation	47 (14.9%)
Aneurysm treatment	
- Clipping	170 (54%)
- Coiling	145 (46%)
WFNS	
- Grade I	114 (36.2%)
- Grade II	37 (11.7%)
- Grade III	34 (10.8%)
- Grade IV	64 (20.3%)
- Grade V	66 (21%)
Fisher	
- Grade 1	0 (0%)
- Grade 2	35 (11.1%)
- Grade 3	108 (34.3%)
- Grade 4	172 (54.6%)

*WFNS*, World Federation of Neurosurgical Societies

### Comorbidities and illness severity score

**Fig. 2:**
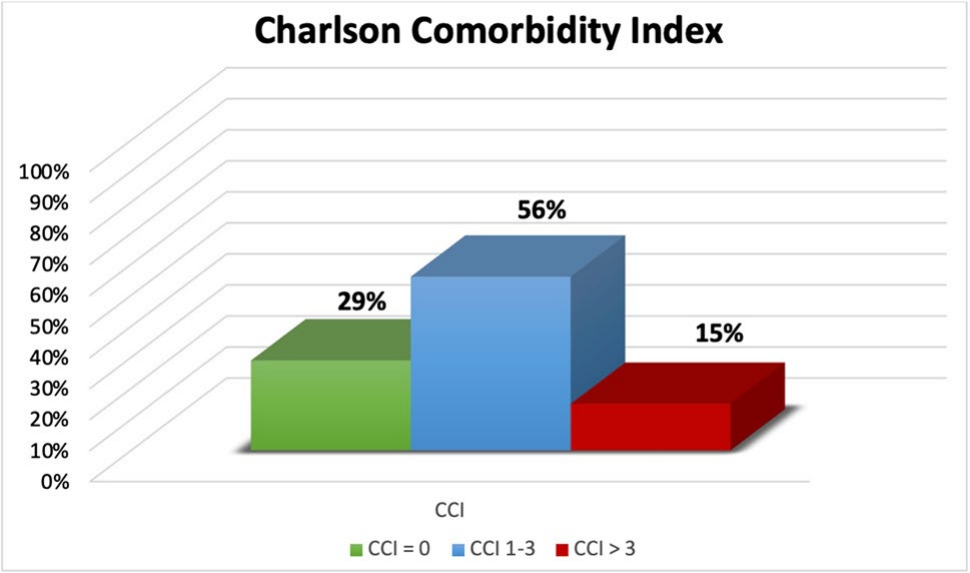
Distribution of Charlson comorbidity index (CCI) within the entire study population with comorbidities found in 71% of patients and significant comorbidities (CCI > 3) in 15% of patients

**Fig. 3:**
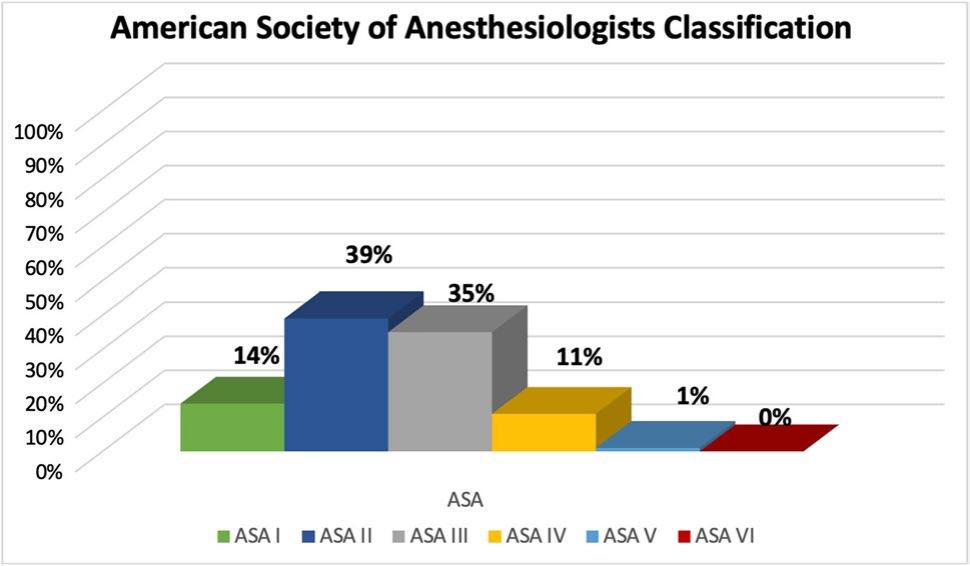
American Society of Anesthesiologists (ASA) classification distribution within the entire study population with significant physical status impairment (ASA > 3) found in 12% of patients

At least one comorbidity was present in 73.3% (231/315) of all patients (Fig. 2). The most common comorbidity in the study population was arterial hypertension (34%), followed by nicotine abuse (11.9%), cardiac disease (11.4%), and thyroid gland dysfunction (10.5%). According to CCI, in 29% (91/315), no comorbidities were found, 56% of the patients had a CCI 1–3, and 14.6% (46/315) of the patients had significant comorbidity with CCI > 3. Substantial disease burden with impairment of physical performance defined as ASA > 3 had 12% (38/315) (Fig. 3). The median SAPS II in the study cohort was 33 (95% CI 29–35). A SAPS II cutoff value of 40 could significantly discriminate patients with good outcome from those with poor outcome (mRS > 3). Mean values of the SAPS II score in the first 14 days after ictus are summarized in Table 2, showing persistent high values in patients with initially high values and persistent low values in patients with initially low values.

**Table 2:** Mean SAPS II score in the first 14 days after ictus in the patient group with initially high SAPS II score (≥ 28) and in the patient group with initially low SAPS II score (< 28)

SAPS II	Day 1	Day 2	Day 3	Day 4	Day 5	Day 6	Day 7	Day 8	Day 9	Day 10	Day 11	Day 12	Day 13	Day 14
Patients with low SAPS II (mean ± SD)	27.1 ± 10.6	26.5 ± 8.9	24.8 ± 8.7	24.1 ± 8.7	24.1 ± 8.6	24.3 ± 9.1	24.1 ± 8.9	24.5 ± 8.8	23.8 ± 8.7	24.1 ± 9.1	24.6 ± 8.8	24.9 ± 9.6	24.7 ± 8.9	24.9 ± 8.9
Patients with high SAPS II (mean ± SD)	33.8 ± 10.8	32.7 ± 8.3	30.1 ± 8.9	30.4 ± 7.5	30.9 ± 8.8	31.2 ± 8.8	31.4 ± 9.6	31.2 ± 8.2	30.2 ± 8.2	31.2 ± 8.2	29.9 ± 9.5	29.5 ± 9.9	28.7 ± 8.5	29.9 ± 8.4

### Early predictors of functional outcome and in-hospital mortality

**Fig. 4:**
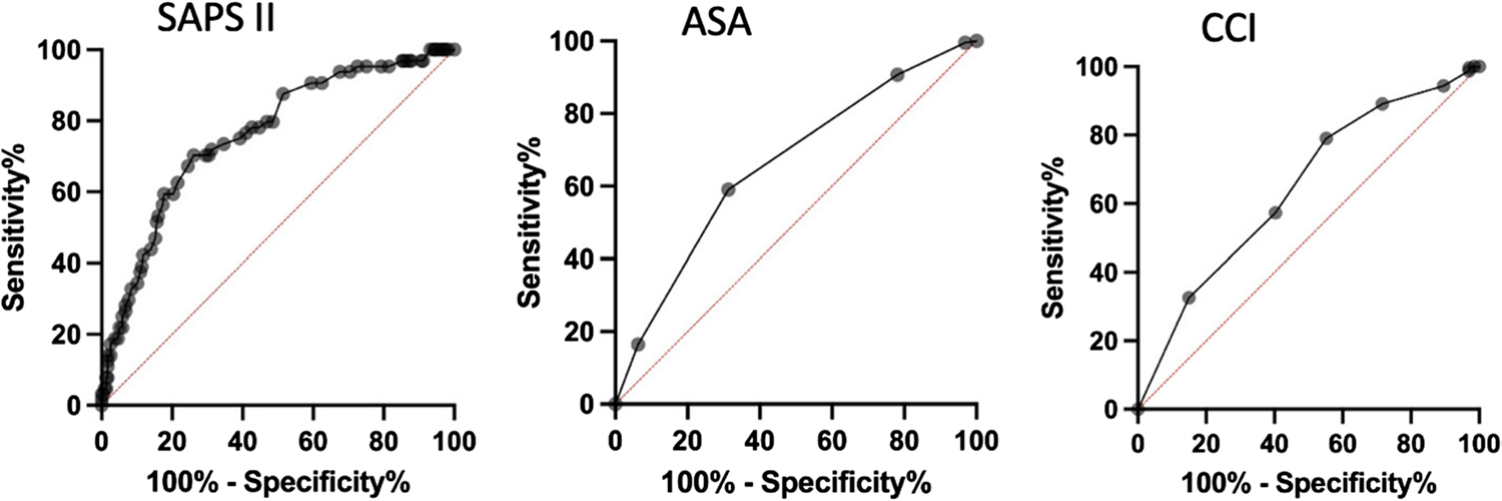
ROC analysis showing the highest discrimination power of good and poor outcome (mRS > 3) for SAPS II with a cutoff value of 40 (AUC 0.76), followed by ASA with a cutoff value of 3 (AUC 0.65) and CCI with a cutoff value of 3 (AUC 0.64)

The mean mRS at 3-month follow-up in the entire patient cohort was 1.6 ± 1.9 (median 1). Poor outcome (mRS > 3) had 21.2% (67/248) of all patients. All three scores, CCI (*r* = 0.2230, *p* < 0.0001), ASA (*r* = 0.2420, *p* < 0.0001), and SAPS II (*r* = 0.3002, *p* < 0.0001), correlated significantly with functional outcome at 3-month follow-up. The median CCI in the patient group with poor outcome was 2 (95% CI 1–3) and 1 (95% CI 0–2) in the patient group with good outcome (*p* < 0.0001). The median ASA in the patient group with poor outcome was 3 (95% CI 3–3) and 2 (95% CI 2–2) in the patient group with good outcome (*p* < 0.0001). The patient group with poor outcome had a significantly higher median SAPS II (44, 95% CI 37–49) compared to the patient group with good outcome (29, 95% CI 28–33), *p* < 0.0001. The best discrimination of patients with good and poor outcome showed SAPS II (AUC 0.76), followed by ASA (AUC 0.65) and CCI (AUC 0.64) (Fig. 4). The in-hospital mortality rate in the entire patient population was 6% (18/315), with a median hospital stay until death of 8.5 (range 2–19) days. In all patients, a decision for termination of treatment was made. The reasons for termination of treatment consequently leading to death were as followed: septic shock with subsequent multiple organs failure (7/18 patients, 39%), diffuse brain swelling with refractory increase in intracranial pressure (7/18 patients, 39%), cardiopulmonary resuscitation at aSAH manifestation resulting into hypoxic brain damage (2/18 patients, 11%), and massive re-bleeding prior to aneurysm occlusion with herniation and infaust prognosis (2/18 patients, 11%). The median CCI in the patients who died (3, 95% CI 1–3) in the hospital was significantly higher compared to the median CCI of the survivors (1, 95% CI 1–2), (*p* = 0.03). The median ASA was also statistically significant higher in the patients who died in the hospital compared to those who survived (2 vs. 3, *p* = 0.02). The median SAPS II score was significantly higher in patients who died in hospital compared to the survivors (44 vs. 31, *p* < 0.0001). In the multivariate analysis, independent predictors of poor outcome were age at diagnosis and SAPS II ≥ 40, whereas CCI, ASA, delayed infarction, and WFNS grading were no independent outcome predictors (Table 3). Considering the WFNS grading, 13% of the patients with lower WFNS grade (I–III) and 33% of the patients with higher WFNS grade (IV, V) had a poor outcome. Patients with high WFNS have a three-fold higher risk for poor outcome compared to patients with lower WFNS (OR 3.149, 95% CI 1.568–6.197). A higher WFNS grade was able to predict poor outcome with a sensitivity of 71%, specificity of 56%, positive predictive value of 32%, and a negative predictive value of 87%, *p* < 0.002. When additionally considering SAPS II to the WFNS grade, poor outcome could be predicted with higher sensitivity (81%), higher specificity (63%), higher positive predictive value (47%), and higher negative predictive value (89%), *p* < 0.0001. Patients with lower WFNS and SAPS II < 40 had a sevenfold lower risk for poor outcome compared to patients with higher WFNS grade and SAPSI I ≥ 40 (OR 7.175, 95% CI 3.387–15.41). Three early prognostic groups concerning functional outcome can be defined according to WFNS and SAPS II (cutoff 40): prognostic group 1 with lower WFNS (I–III) and SAPS II < 40 (poor outcome in 11%, mortality rate 3%), prognostic group 2 with lower WFNS (I–III) but higher SAPS II ≥ 40 (poor outcome in 17%, mortality rate 5%), prognostic group 3 with higher WFNS (IV–V) but lower SAPS II < 40 (poor outcome in 20%, mortality rate 8%), and a prognostic group 4 with higher WFNS (IV–V) and higher SAPS II ≥ 40 (poor outcome in 47%, mortality rate 12%). The functional outcome and in-hospital mortality rate dependent on the WFNS grade and the SAPSII score are listed in Table 4. Extracerebral organ dysfunctions have contributed to 88% (95% CI 85–100) of all points included in the overall SAPS II calculation, whereas the remaining 12% (95% CI 5–16) originate from the assigned GCS points.

**Table 3:** Predictors of poor functional outcome (mRS > 3)

Variables	Odds ratio	95% CI	*p*-value
Age at diagnosis	1.038	1.014–1.064	0.002*
High WFNS (IV, V)	0.8065	0.4067–1.572	0.53
Delayed infarction	2.176	0.9790–4.774	0.05
CCI > 3	0.4101	0.1254–1.113	0.10
ASA > 3	0.6396	0.1862–1.842	0.43
SAPS II ≥ 40	6.064	3.196–11.95	< 0.0001*

Multivariate logistic regression analysis. *CCI*, Charlson comorbidity index; *ASA*, American Society of Anesthesiologists; *SAPS*, simplified acute physiology score; *WFNS*, World Federation of Neurosurgical Societies

The significant *p* values were markd with *

**Table 4:** Defining prognostic groups by additional consideration of SAPS II to WFNS grading

Prognostic groups functional outcome	Good outcome (mRS ≤ 3)	Poor outcome (mRS > 3)	Mortality rate
Group 1: Lower WFNS (I-III) + lower SAPS II < 40	89%	11%	3%
Group 2: Lower WFNS (I-III) + higher SAPS II ≥ 40	83%	17%	5%
Group 3: Higher WFNS (IV-V) + lower SAPS II < 40	80%	20%	8%
Group 4: Higher WFNS (IV-V) + higher SAPS II ≥ 40	53%	47%	12%

*WFNS*, World Federation of Neurosurgical Societies; *SAPS*, simplified acute physiology score; *mRS*, modified Rankin scale

## Discussion

The course of aSAH can be complicated not only by neurological adverse events such as re-bleeding, hydrocephalus, progressive brain edema, or delayed infarction but also by non-neurological predominantly cardiological and pulmonary complications [10, 33]. Extracerebral organ dysfunction has been reported to have a substantial influence on outcome and should be responsible for up to 40% of deaths after aSAH [9, 29]. The presence of comorbidities bears a risk to additionally aggravate the course of acute diseases [26]. Despite growing evidence about interactions of aSAH with the function of multiple organ systems, comorbidity and illness severity scores are not widely used for early prognosis estimation in aSAH patients. In this observational study, we aimed to address this relevant question and to shed light on the prognostic role of comorbidities and illness severity scores in a large consecutive aSAH patient population treated according to the same management protocol. Three already existing and frequently used scoring systems were directly compared to evaluate the impact of comorbidities, comorbidity-associated functional impairment, and acute organ dysfunction within the first 24 h after admission on in-hospital mortality and functional outcome in aSAH patients. According to the results of our study, CCI and ASA do not seem to have sufficient discrimination power as prognostic factors for early separation of patients with high odds to have a good prognosis from those, who will probably experience poor outcome after aSAH. The explanation for these findings might be the only small portion of patients with relevant comorbidities (CCI > 3 was found in 15%), and substantial physical performance status impairment (ASA > 3) had only 12% of all patients. Boogaards et al. have previously evaluated the CCI in the setting of aSAH and could not find any correlation of CCI with outcome in aSAH patients [3]. The reason for this finding might be the insufficient consideration of comorbidity-related physical performance impairment by the CCI. Since a younger patient population is affected by aSAH compared to other stroke types, not only the estimation of the probability of survival but also the probability of achieving good functional outcome without disability is substantial in aSAH patients. The ASA classification considers not only the presence of comorbidities but also integrates the functional impairment due to the present comorbidities. Therefore, it is deemed to circumvent this limitation, which was the rationale to evaluate the prognostic value of the ASA classification in aSAH patients. To the best of our knowledge, this is the first study evaluating the ASA classification for early outcome estimation in an aSAH population in comparison to CCI. While all three scores significantly correlated with functional outcome in univariate analysis, only SAPS II was an independent predictor of outcome in multivariate analysis. SAPS II also demonstrated a higher discrimination power between good and poor functional outcome, compared to CCI and ASA. In contrast to CCI and ASA, the SAPS II score might have provided a better depiction of the systemic reaction induced by the aSAH with subsequent extracerebral organ dysfunctions, which would justify an integration of SAPS II score into early prognostication tools in aSAH patients. SAPS II was an independent predictor not only of in-hospital mortality but also of functional outcome, providing the highest contribution to a reliable early outcome estimation of aSAH patients in our study. SAPS II contributed to better early prognosis estimation compared to WFNS grading alone. The consideration of SAPS II within the first 24 h after ictus seems to be useful for identifying good grade aSAH patients with a risk for poor outcome as well as poor grade aSAH patients with higher probability for good outcome, which is of great clinical relevance during the acute management of aSAH patients. Since GCS is part of SAPS II score calculation and represents the basis of WFNS grading at the same time, it was important to separately evaluate the contribution of GCS and of extracerebral organ dysfunction to the overall SAPS II calculation. Extracerebral organ dysfunctions contributed on average to 88% of the overall score calculation, and the remaining points came from GCS. These findings also support the importance of considering extracerebral organ dysfunction for early outcome estimation after aSAH. Several illness severity scores are currently available for prognostic evaluation in intensive care unit patients such as SOFA (Sequential Organ Failure Assessment), APACHE (Acute Physiology and Chronic Health Evaluation), and MPM (Mortality Probability Model) [21, 24, 32]. While the SOFA score is mainly focused on sepsis-related organ failure assessment, the APACHE score and the MPM have been primarily developed for mortality estimation. A common denominator of all these scores is the consideration of multiple physiological parameters representative for the function of almost all organ systems. However, they differ concerning the complexity of their calculation, which is one reason why these scores are not routinely used in all hospitals. Since SAPS II score has been prospectively calculated as part of the clinical practice in all patients treated at the intensive care unit at our institution, these data were available for a prognostic evaluation in aSAH patients. On the contrary, SOFA, APACHE, and MPM are not routinely applied at our institution, which prohibited their consideration for the analysis of this study.

### Early prediction of in-hospital mortality and functional outcome after aSAH

Over the last years, a continuous decrease of in-hospital mortality after aSAH has been reported in the literature [12, 16, 31]. La Pira and colleagues assessed the in-hospital mortality in a large patient population with aSAH from 1985 to 2014 and reported an in-hospital mortality of 22.6% in the time 1985–1994 compared to 16.7% in the time 2005–2014 [12]. The in-hospital mortality in our study population encompassing the treatment time 2012–2020 was 6% that is notably lower compared to the study population in the publication from La Pira et al., and at the same time underlines a continuous decline in in-hospital mortality in aSAH patients over time. Several causes of in-hospital death have been identified so far such as re-bleeding, refractory cerebral edema, or the development of cerebral or extracerebral complications [1], which was in line with the identified causes of death in our study. The reduction in re-bleeding rates by early aneurysm repairment within 48–72 h after aneurysm rupture is deemed to be one the most relevant contributors to reduced case fatality after aSAH alongside with treatment advances in intensive care medicine and the diagnostics and therapy of delayed ischemic complications [31]. However, not all mortality predictors are available within the first 24 h after ictus, hence, not equally allowing an early prognosis estimation. Lee et al. developed a simple risk stratification score (HAIR score) including aSAH-related independent predictors of in-hospital mortality (Hunt and Hess grade, age, intraventricular hemorrhage, and re-bleeding within 24 h after ictus) analogue to the intracerebral hemorrhage score (Hemphill ICH score) and demonstrated an association of a higher HAIR score with higher in-hospital mortality rate [13]. The research of the last years led to an increasing recognition of a relevant impact of extracerebral complications and organ dysfunctions on in-hospital mortality after aSAH additional to cerebral hemorrhage-related complications, which requires a consideration during early outcome estimation after aSAH. Several scores based on multiple parameters representing organ functions have been developed for mortality prediction in ICU patients, whereby only limited data exist on the prognostic role of these scores in aSAH patients. Most of these scores are complex involving a substantial amount of data, which is probably the main reason why these scores had not been widely used in clinical practice. One important aspect may be the time point of prognosis estimation. Most studies have focused on calculating the score at admission, as the earliest time point. Rubbert et al. have identified several frequently used prognostic parameters available at admission such as Fisher and WFNS grading, age, early insertion of external ventricular drainage, and the mean transit time on early computed tomography perfusion [22]. On the other side, other authors have pursued the approach of performing multiple calculations at different time points allowing the assessment of score development over time. Zeiler et al. have prospectively evaluated the FOUR (Full Outline UnResponsiveness) score outcome prediction in aSAH patients applying the score at admission, on day 7, and on day 14 after ictus [35]. While the score calculation at admission and on day 7 had comparative prognostic value, the score on day 14 was only associated with 6-month outcome [35]. The calculation of the SAPSII score over 14 days showed persistently high values in the patients with high scores at admission. Additionally, to the established grading scores, there are an increasing number of recent publications using machine learning, which could play an important role in the future [8, 18].

The decision of treatment termination on the intensive care unit is crucial aspect, which needs to be considered when evaluating predictors of in-hospital mortality, because it might introduce a bias. Indeed, in all patents of our study, who died in the hospital, a decision for treatment termination was made. In all cases, an infaust prognosis was anticipated, whereby the proportions of cerebral and extracerebral dysfunctions contributing to this decision were equally distributed. This underlines the need for a prognostication tool, which includes cerebral and extracerebral dysfunctions. The SAPS II score fulfills these criteria and was an independent predictor of in-hospital mortality in our study with the best discrimination validity. Schuiling et al. have evaluated the SAPS II score in 148 aSAH patients treated between 2000 and 2002 for its prognostic value for DCI and clinical outcome. They found a significant correlation of higher SAPS II score with DCI and outcome, but the discrimination power for DCI was very low with an AUC of 0.52 [25]. On the other hand, the discrimination power concerning clinical outcome was much better with AUC of 0.85, which was also slightly higher than the discrimination power in our study with AUC of 0.76. In contrast to the findings of Schuiling et al., we found no significant correlation of CCI, ASA, or SAPS II with DCI. In the multivariate analysis, DCI did not reach statistical significance for the prediction of functional outcome. A possible explanation might be the fact that not every delayed infarction resulted into neurological deficits directly contributing to a poorer mRS. In a more recently published study by Czorlich et al., SAPS II was evaluated for the prediction of in-hospital mortality in aSAH patients. Additionally, the authors aimed to improve the discrimination power by adding further independent mortality predictors (history of chronic headache, intake of anticoagulation), and demonstrated a slight increase in the AUC from 0.83 to 0.86 [6]. However, the reported in-hospital mortality rate in the publication of Czorlich et al. was substantially higher compared to the mortality rate in our study (6% vs. 18%), and Czorlich et al. have used a lower cutoff value for SAPS II than the calculated cutoff value in our study (30 vs. 40), which might have contributed to the different discrimination power of SAPS II. Finally, the treatment period also differed in both studies (2010 to 2014 vs. 2012 to 2020), which might have had an impact on outcome as well. This highlights the importance of comparing the scores in similar populations. Taking into consideration the results of our study, a score representing the acute cerebral and extracerebral organ dysfunction within the first 24 h after aneurysm rupture seems to be the best early prognostication tool in aSAH patients. An additional consideration of SAPS II to SAH-related WFNS score contributed to even better discrimination of poor and good outcome and facilitated the definition of three prognostic groups with early prognosis estimation in aSAH patients with 24 h after ictus. This approach might be the next step towards developing a comprehensive disease-specific illness severity score for aSAH patients. There is an increase in publications considering also neuroinflammatory parameters for an early prognosis estimation after aSAH [17, 36]. A recently published article reports about a new comprehensive score (the TAPS score) including not only well-established parameters such as WFNS or Fisher grading but also considering early brain injury indicators such as white blood cell count, which have been demonstrated to facilitate a good 90-day outcome prediction in aSAH patients [15].

A better understanding of the brain-systemic interactions could contribute to a more reliable identification of parameters that merit a consideration in an illness severity score for patients with aSAH. The additional consideration of SAPS II seems to be helpful for identifying patients with lower WFNS grade with higher probability for poor outcome or in-hospital mortality as well as to identify patients with higher WFNS with a probability for good outcome which could alleviate the treatment decision-making during the acute management of aSAH patients.

### Limitations of the study

A substantial limitation of our study is the retrospective data analysis. We cannot exclude an incomplete assessment of comorbidities, which might have had led to different findings concerning the prognostic role of CCI and ASA. The impact of specific cerebral and extracerebral complications during the course of disease and their specific impact on outcome were not separately evaluated in our study, which is another limitation of the retrospective analysis. The double consideration of GCS in the WFNS grading and the calculation of the SAPS II score needs to be acknowledged as a limitation during data interpretation, which could be possibly overcome by developing an aSAH-specific score including parameters representing cerebral and extracerebral organ dysfunctions. Our study was primarily focused on assessing established scores rather than on developing a new score. Hence, this objective should be pursued in a future study.

## Conclusion

The disease burden encompassing the comorbidities and organ functions at aSAH significantly correlated with functional outcome at 3-month follow-up and with in-hospital mortality in this retrospective observational study. The results of our study support the hypothesis that aSAH should be regarded as a systemic disease and highlight the necessity of integrating multiple organ functions in the tools for early prognosis estimation after aSAH. An additional consideration of SAPS II might be supportive to identify good grade aSAH patients (WFNS grade I–III) with higher probability of poor outcome as well as poor grade aSAH patients (WFNS grade IV, V) with higher probability of good outcome and facilitate a better guidance of treatment decisions during the acute management of aSAH patients. The development of a comprehensive disease-specific illness severity score for aSAH with consideration of cerebral and extracerebral dysfunctions with higher discrimination power is needed for a reliable early prognosis estimation in aSAH patients.

## Data Availability

All available data is presented in the manuscript.
